# Video-assisted thoracoscopic treatment as two-day surgery for lung neoplasms: a propensity-matched analysis

**DOI:** 10.1186/s12885-022-09938-x

**Published:** 2022-07-30

**Authors:** Guofei Zhang, Junqiang Fan, Zipu Yu, Ying Chai, Sai Zhang, Ming Wu, Gang Shen

**Affiliations:** Department of Thoracic Surgery, the Second Affiliated Hospital of the Zhejiang University School of Medicine, 88 Jiefang Road, Hangzhou, 310009 China

**Keywords:** Thoracic surgery, Video-assisted, Enhanced recovery after surgery, Lung neoplasms, Postoperative complications, Propensity matching

## Abstract

**Background:**

Enhanced recovery after surgery programs have reduced complications and shortened hospital stays after lung resection. This study aimed to determine whether video-assisted thoracoscopic surgery performed as a two-day surgery for lung neoplasms was safe and cost-effective.

**Methods:**

This retrospective, propensity-matched, cohort analysis was conducted from January 2020 to August 2020. Among 959 patients who underwent video-assisted thoracoscopic surgery, 739 underwent inpatient surgery and 220 underwent two-day surgery. Propensity-matched analysis, incorporating preoperative variables, was used to compare postoperative complications, post-discharge follow-up results, and hospitalization costs between the groups.

**Results:**

Propensity matching estimated 218 patients in each group. The mean length of hospital stay was shorter in the two-day surgery group (2.17 ± 0.89 days) than in the inpatient surgery group (6.31 ± 2.13 days) (*P* < 0.001). Delayed removal of chest tubes accounted for over half of the delayed discharges in the inpatient (17 [54.8%]) and two-day surgery (13 [65.0%]) groups. The postoperative pneumonia/atelectasis incidence was lower in the two-day surgery group than in the inpatient surgery group (*P* = 0.032). The two-day surgery group patients were readmitted to the hospital due to massive pleural effusion, pneumothorax, fever, severe chest pain, and physical weakness. The mean total hospitalization cost in the two-day surgery group was lower than that in the inpatient surgery group (¥ 33,926.1 versus ¥ 38,422.7, *P* < 0.001). Basic medical, nursing, drug, laboratory-related, and nonsurgical consumable costs in the two-day surgery group were significantly reduced.

**Conclusions:**

Two-day surgery is a safe, feasible, and cost-effective procedure for selected patients with lung neoplasms when combined with accurate preoperative evaluations, successful intraoperative assessments, and effective postoperative health care guidance.

**Supplementary Information:**

The online version contains supplementary material available at 10.1186/s12885-022-09938-x.

## Background

Enhanced recovery after surgery (ERAS) programs, which are now widespread, represent a multimodal approach to promote early recovery after surgical procedures; this approach aims to lessen surgical stress responses, reduce complications, lower medical costs, and promote patient recovery by optimizing a series of perioperative management measures [1]. ERAS was first introduced for colorectal cancer surgery in the late 1990s [2]. Regarding thoracic surgery, particularly for the surgical treatment of lung neoplasms, ERAS data are limited [3, 4]. A systematic meta-analysis from seven randomized controlled trials on ERAS adoption for lung cancer surgery revealed a clinical superiority similar to that of the colorectal cancer ERAS program for the effective control of postoperative morbidity and length of hospital stay (LOS) [5].

Day surgery, also known as same-day surgery, is the admission of selected patients to a hospital or surgery center for a planned surgery or procedure with the expectation that the patient is discharged on the same day or within 24 h [6]. Day surgery is the most effective form of health care provision because it ensures public resource savings [7]. Good evidence is currently available to support a day-case approach for various thoracic procedures, both diagnostic and therapeutic, such as lung biopsy, thoracic sympathectomy, and pneumothorax surgery [8, 9]. However, despite various minimally invasive approaches for lung neoplasms and the general standardization of the perioperative phase, the clinical outcomes of video-assisted thoracoscopic surgery (VATS) as day surgery for lung neoplasms are limited. This lack of clinical outcomes may be related to the fact that anatomical lung resection with or without mediastinal lymph node dissection remains an invasive and traumatic procedure associated with considerable postoperative, mainly cardiopulmonary, morbidity.

Maruyama et al. [10] first proposed VATS as a two-day surgery for spontaneous pneumothorax in 2000. They found that the two-day surgery model is a safe and cost-effective procedure for spontaneous pneumothorax, which is consistent with the requirements of rapid recovery after lung surgery. However, the feasibility and safety of the two-day surgery approach in lung neoplasm surgery have not been demonstrated. We began using VATS as a two-day surgery for lung neoplasms in April 2020. This study aimed to assess the clinical outcomes and cost-effectiveness of two-day surgery performed for lung neoplasms.

## Methods

### Study design and patients

We performed this retrospective propensity-matched analysis that included 739 patients with pulmonary neoplasms who underwent inpatient VATS and 220 who underwent two-day VATS at the Second Affiliated Hospital of the Zhejiang University School of Medicine from January 2020 to August 2020. The inclusion criteria were as follows: age < 90 years; computed tomography (CT) suggesting the presence of lung neoplasms that required surgery; classified as American Society of Anaesthesiologists physical status class I or II; and complete clinical data and signed informed consent forms. All patients treated with two-day surgery underwent lobectomy, segmentectomy, or wedge resection. Therefore, for a more accurate comparison, patients who underwent the following procedures were excluded from the inpatient surgery group (ISG): conversion to open thoracotomy, complex sleeve lobectomy, surgery for other diseases during hospitalization, and robot-assisted VATS. In addition, patients who were transferred to multiple clinical departments were excluded. Data on clinical parameters, including patient age, sex, type of neoplasm, surgical procedure performed (wedge resection, segmentectomy, or lobectomy), LOS (preoperative, postoperative, and total), postoperative and post-discharge events, and hospitalization costs, were collected from medical and follow-up records. The characteristics of the patients are detailed in Table 1.

**Table 1:** Characteristics of patients in the inpatient surgery and two-day surgery groups

	Unmatched patients					Propensity-matched patients				
	ISG		TSG			ISG		TSG		
	717		219			218		218		
Characteristics	n	%	n	%	*P*	n	%	n	%	*P*
Sex										
Male	285	39.7	79	36.1	0.329	69	31.7	78	35.8	0.362
Female	432	60.3	140	63.9		149	68.3	140	64.2	
Age, y										
≤ 30	19	2.6	15	6.8	< 0.001	11	5.0	14	6.4	0.071
> 30, ≤ 49	166	23.2	74	33.8		74	33.9	74	33.9	
> 50, ≤ 70	438	61.1	123	56.2		113	51.8	123	56.4	
> 70	94	13.1	7	3.2		20	9.2	7	3.2	
Operative procedures										
Wedge	129	18.0	64	29.2	< 0.001	76	34.9	63	28.9	0.355
Segmentectomy	236	32.9	91	41.6		79	36.2	91	41.7	
Lobectomy	352	49.1	64	29.2		63	28.9	64	29.4	
Neoplasm										
Benign	67	9.3	15	6.8	0.004	20	9.2	15	6.9	0.073
Preinvasive	40	5.6	26	11.9		13	6.0	26	11.9	
Malignant	610	85.1	178	81.3		185	84.9	177	81.2	

*ISG* Inpatient surgery group, *TSG* Two-day surgery group

### Two-day surgery group

We performed VATS as a two-day surgery in April 2020. All two-day surgery group (TSG) patients underwent preliminary screening at the outpatient clinic; information on age, underlying diseases, especially respiratory diseases, and previous surgical history were obtained. The two-day VATS approach was recommended for suitable patients. However, the choice of either two-day VATS or inpatient VATS depended on patient preference. Next, preoperative conditions, such as smoking cessation and control of comorbidities, including diabetes and hypertension, were considered. All preoperative tests were completed before hospital admission. The second assessment was performed by reviewing examination results to determine whether there were serious cardiovascular or cerebrovascular diseases and other serious diseases or unstable medical conditions. Next, patients were confirmed for enrollment in the TSG and were contacted by telephone to discuss precautions for surgical procedures. The patients in the TSG were admitted on the day of surgery and were discharged within 48 h after the operation.

### Inpatient surgery group

Patients in the ISG underwent preliminary screening in the outpatient clinic, followed by preoperative examinations and evaluations, and were admitted 1–5 days before surgery. Patients received respiratory preparation and education prior to surgery.

### Surgical techniques

The surgical procedures have been described in detail previously [11–13]. Briefly, all surgical procedures were performed under general anesthesia with double-lumen intubation. Patients were placed in the full lateral decubitus position. Thoracoscopic surgery was performed via a three-incision or single-incision approach. Wedge resection, segmentectomy, or lobectomy was performed according to the location and size of lesions, frozen section, and pulmonary function, among others. Mediastinal lymph node dissection was performed if needed. A single 24-F chest tube, which was connected to a negative-pressure drainage bottle, was inserted in the incision up to the upper border of the thoracic cavity prior to closure of the port site. Concurrently, a 14-G fine tube that was connected to a drainage bag was placed in the intercostal space inferior to the incision.

### Postoperative treatment and follow-up

Prophylactic antibiotics and analgesics were administered postoperatively. For patients in the TSG, a chest radiograph was obtained on the first postoperative day. If no air leakage was observed and the lung was inflated, the 24-F chest tube was removed. The 14-G fine tube was removed the next morning, and the patient was discharged. In the ISG, the 24-F chest tube was removed on the second day when there was no air leakage. The 14-G fine tube was removed prior to discharge.

All patients received a follow-up telephone call 1 week after discharge and an outpatient review 3 weeks after discharge. The follow-up included questions and assessments related to their general condition, clinical symptoms, pain visual analog pain scale (VAS) scores, fever (> 38.5 °C), and readmission. Outpatient chest radiographs or CT images were evaluated for the presence of pleural effusion, atelectasis, pneumothorax, or inflammation.

### Statistical analysis

Descriptive statistics were used to characterize demographic and clinical characteristics. Categorical variables are presented as frequencies and proportions, and they were compared using the chi-square test or Fisher’s exact test, as appropriate. Normally distributed continuous variables are expressed as the mean ± standard deviation, and they were compared using a Student’s *t*-test. Continuous variables not following a normal distribution are expressed as the median [interquartile range] and were compared using the Wilcoxon rank-sum test.

To compare postoperative complications, post-discharge events, and hospitalization expenses between the TSG and ISG, a one-to-one matching analysis between the two groups on the basis of the propensity score was performed to minimize bias owing to the non-random allocation of treatments among patients [14]. Propensity scores were estimated using a logistic model that included the following variables: age, sex, type of neoplasm, and surgical procedure. The propensity score summarizes these features in a single variable that can be included in analyses comparing postoperative outcomes and cost-effectiveness between the two groups. Each patient who underwent thoracoscopic lung resection in the TSG was matched with a patient in the ISG, with the closest estimated propensity score on the logit scale.

Differences were considered statistically significant when the probability value was below 0.05. Data analysis was performed using the Statistical Package for Social Sciences software (version 26.0, Chicago, IL).

## Results

### Baseline clinical characteristics

**Fig. 1:**
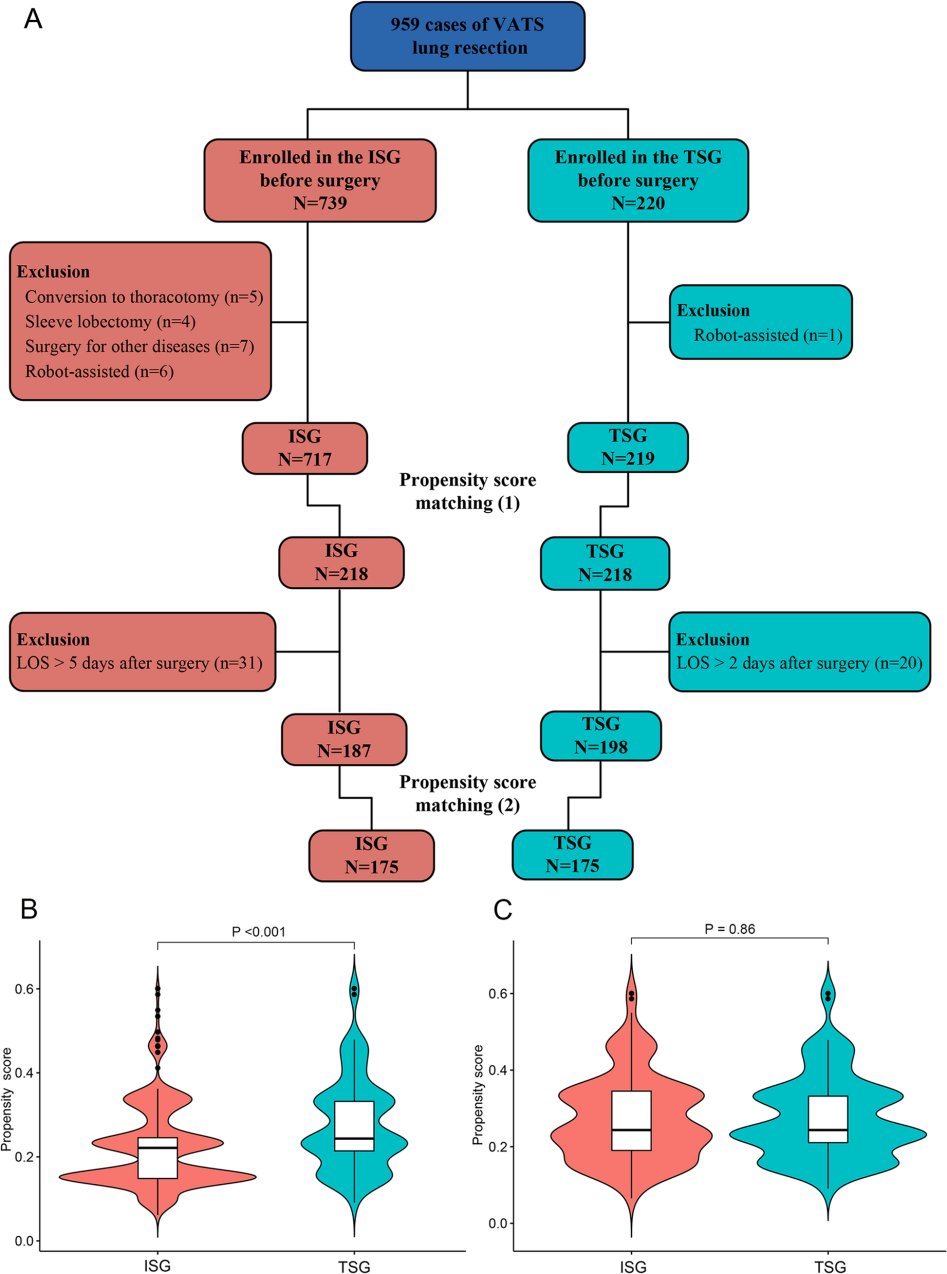
Study flow diagram (**A**) and distribution of propensity scores in the unmatched patients (**B**) and matched patients (**C**). ISG: inpatient surgery group; TSG: two-day surgery group; VATS: video-assisted thoracoscopic surgery; LOS: length of stay

There was no operative mortality in either group. Twenty-three patients met the exclusion criteria and were excluded from the study. Thus, 936 patients were included in the study: 717 in the ISG and 219 in the TSG. The patient selection flowchart is shown in Fig. 1a. In the unmatched cohort (Table 1), there was a significant difference between the groups with respect to age, operative procedure, and type of neoplasm. Therefore, we performed one-to-one propensity score matching to compare clinical outcomes and hospitalization expenses between the ISG and TSG. When propensity score matching was used for variables, including age, sex, operative procedure, and type of lung neoplasm, the ISG and TSG were well matched (218 patients each) without significant differences in those variables (Fig. 1b, c).

### Length of hospital stay

The median LOS in the TSG was significantly shorter than that in the ISG (2.17 ± 0.89 days versus 6.31 ± 2.13 days, *P* < 0.001). Preoperative LOS in the ISG was 2.6 ± 1.44 days, while that in the TSG was 0 days (*P* < 0.001); postoperative LOS in the ISG and TSG was 3.75 ± 1.61 days and 2.17 ± 0.89 days, respectively (*P* < 0.001).

### Perioperative complications and post-discharge events

A detailed analysis of the complications that resulted in delayed discharge in the propensity-matched ISG and TSG is shown in Table 2. Interestingly, the incidence of postoperative pneumonia/atelectasis in the TSG was significantly lower than that in the ISG (*P* = 0.032).

**Table 2:** Postoperative complications resulting in delayed discharge in the propensity-matched inpatient surgery and two-day surgery groups

Events	ISG	TSG	*P* value
	218	218	
Delayed discharge	31 (14.2%)	20 (9.2%)	0.068
Prolonged air leakage	11 (5.0%)	10 (4.8%)	0.823
Abnormal pleural effusion^a^,	6 (2.8%)	3 (1.4%)	0.312
Fever, ≥ 38.5 °C	3 (1.4%)	2 (0.9%)	0.653
Chest pain, ≥ 5	1 (0.5%)	1 (0.5%)	1.000
Pneumonia/atelectasis^b^	7 (3.2%)	1 (0.5%)	0.032
Abnormal function of other organs^c^	3 (1.4%)	3 (1.4%)	1.000

*ISG* Inpatient surgery group, *TSG* Two-day surgery group

^a^ Including pleural effusion > 500 ml/24 h, hemothorax and chylothorax

^b^ Need antibiotics

^c^ Including arrhythmia, cerebrovascular abnormalities, liver function abnormalities, among others, that require medication

All patients received a follow-up telephone call 1 week after discharge. Among the propensity score-matched patients, none had serious events requiring emergency treatment. Among the scheduled discharge patients, the number of patients requiring readmission was eight (4.3%) in the ISG and nine (4.5%) in the TSG (Table 3).

**Table 3:** Reasons for readmission of scheduled discharged patients

Events	ISG	TSG	*P* value
	187	198	
Readmission	8 (4.3%)	9 (4.5%)	0.898
Fever^a^	1	3	
Pleural effusion	5	1	
Pneumothorax	0	1	
Pain	0	1	
Other	2^b^	3^c^	

*ISG* inpatient surgery group, *TSG* two-day surgery group

^a^ Fever > 38.5 °C

^b^ Two patients were admitted to the hospital because of intestinal obstruction

^c^ Admission to the local hospital because of weakness

Within 3 weeks after discharge, 183 (83.9%) patients in the ISG and 196 (89.9%) patients in the TSG underwent outpatient chest radiography or CT. The examination results indicated that there was no significant difference between the two groups with respect to the incidence of pleural effusion, pneumothorax, or lung exudate atelectasis (Table 4). According to imaging results and patients’ symptoms, five patients in the ISG and three in the TSG with moderate to large pleural effusion were readmitted to the hospital for treatment. It is worth noting that two patients in the TSG were those with a delayed discharge. Although over 20% of patients had pulmonary exudate/atelectasis, they were not readmitted to the hospital for special treatment but received medication or follow-up treatments.

**Table 4:** Chest radiograph or computed tomography (CT) scan results within 3 weeks after surgery

Events	ISG	TSG	*P* value
	183	196	
Pleural effusion			
Normal to moderate	178 (97.3%)	193 (99.0%)	0.416
Moderate to large^a^	5 (2.7%)	3 (1.0%)	
Pneumothorax			
Normal to small	183 (100%)	195 (99.5%)	0.333
Moderate to large^a^	0 (0)	1 (0.5%)	
Lung effusion or partial atelectasis^b^	41 (22.4%)	49 (25.0%)	0.553

*ISG* Inpatient surgery group, *TSG* Two-day surgery group

^a^ There are obvious symptoms that require further puncture and drainage

^b^ Only medical treatment in outpatients, no readmission

### Hospitalization costs

Table 5 provides the total and component expenses in the ISG and TSG after propensity matching (1:1). The results showed that the mean total expense in the TSG was ¥ 33,926.1, which was significantly lower than that in the ISG (¥ 38,422.7, *P* < 0.001), for a reduction in costs of ¥ 4,496.6 (11.7%). To avoid the influence of postoperative complications on the mean total expense, patients with discharge delays in both the groups were excluded, which resulted in 187 patients in the ISG and 198 in the TSG. After additional propensity score matching, there were 175 matched patients in each group. Similar results to those obtained in the first propensity matching for the total and component expenses were found for the two groups (Additional file 1).

**Table 5:** Total and component costs (¥) after propensity matching (1:1)

Variable	ISG	TSG	Δ ISG-TSG (%)	*P* value
	218	218		
Total costs	38,422.7	33,926.1	4,496.6 (11.7)	< 0.001
General medical service fees	353.6	129.1	224.5 (63.5)	< 0.001
General treatment fees	963.7	500.8	462.9 (48.0)	< 0.001
Nursing fees	674.0	291.7	382.3 (56.7)	< 0.001
Drug costs	4,515.0	3,503.8	1,011.2 (22.4)	< 0.001
Material fees				
For surgery	15,066.8	14,486.5	580.3 (3.9)	0.238
For other treatment	1963.1	1145.4	817.7 (41.7)	< 0.001
For examination	88.4	66.2	22.2 (25.1)	< 0.001
Diagnostic-related fees				
Laboratory	2451.6	1,924.3	527.3 (21.5)	< 0.001
Imaging	2,014.9	1,822.9	192.0 (9.5)	0.145
Surgical expenses	7,859.5	7,948.9	-89.4 (-1.1)	0.384

*ISG* Inpatient surgery group, *TSG* Two-day surgery group

## Discussion

Compared with inpatient surgery, two-day surgery significantly reduced the LOS and reduced hospitalization costs. There was no significant increase in medical events during hospitalization or after discharge. Current evidence demonstrates that morbidity rates, LOS, and health care costs are the three most common evaluation criteria for a standardized ERAS program [1, 4]. The high incidence of lung cancer and its associated mortality rates make this disease one of the most severe public health problems worldwide [15]. How can the utilization of limited medical resources be maximized while still providing high-quality care for patients? The two-day surgery model has the potential to achieve these goals and effectively adhere to ERAS development requirements.

Persistent air leakage after surgery is the main cause of prolonged LOS [16–18]. In our study, the occurrence of persistent air leakage was often related to pleural adhesion or atresia during surgery. Ueda et al. [16] recommend the use of bioprotein glue and naphthalene viol to help reduce postoperative air leakage. Our study showed that the incidence of postoperative pneumonia/atelectasis resulting in delayed discharge in the TSG was significantly lower than that in the ISG, which could be related to the reduction in LOS. For other reasons, such as arrhythmia, lung infections, and other complications, careful evaluation of the patient’s cardiac function, lung function, and cough response before surgery is required.

The two-day surgery model requires optimization of various health care resources in the pre-, intra-, and postoperative settings. Although there was no significant difference in the incidence of readmission between the two groups, some of the patients in the TSG may not have required hospitalization. The main reason is that patients experience insecurity about the recovery process after discharge. The central cause of patients’ feelings of insecurity is that they perceive that it will be difficult to contact the caregiver. The patients experience a lack of professional support and do not know how to obtain help and support [19]. Therefore, in the future, we should provide more effective guidance to patients regarding how to contact relevant health care systems.

Regardless of the use of single-port or multi-port VATS, postoperative pain remains significant and affects quality of life and functional recovery after surgery [20]. In the TSG, one patient who was discharged as planned was readmitted to the hospital owing to severe pain (VAS score > 5). This finding may be attributed to the provision of multimodal analgesia during periods of hospitalization, while only one nonsteroidal anti-inflammatory medication is provided after discharge. Therefore, special attention should be paid to both in-hospital and post-discharge analgesia.

There was no significant difference in the incidence of moderate-to-massive pleural effusion between the two groups of patients on chest radiographs obtained 3 weeks after discharge. Three patients in the TSG had moderate-to-massive pleural effusion and were readmitted for thoracentesis owing to chest tightness. Therefore, we believe that after thoracoscopic lung resection, if there is no air leakage and pleural effusion is considered a general exudation of the pleural cavity after surgery, it is safe to remove the chest tube after 48 h; however, more cases are required to verify this.

As shown in Table 5, surgical consumables and surgical fees accounted for more than half of the total costs, but there was no significant difference between the two groups. However, regarding other main components, there were significant differences between the two groups, especially with respect to laboratory-related fees, drug costs, general treatment fees, and related consumables fees. Moreover, the general medical service fees and nursing costs in the TSG were reduced by more than half compared with those in the ISG, a finding that was related to patient LOS. Therefore, two-day surgery is more cost-effective than inpatient surgery.

Few limitations of this study should be acknowledged. First, the results were based on retrospective data from a review of medical records from a single center. A randomized trial of the ISG and TSG was not performed. Considering ethical and safety concerns, selection bias may have occurred based on patient preferences and close evaluations before patients were enrolled in the TSG at our initial stage. Although propensity matching reduced the selection bias, it was not eliminated. However, with improved results in the initial stage, we can consider prospective randomized controlled studies in the later stage. Future multicenter studies are needed to validate our findings. Second, the medical database does not include surgeon identifiers. Consequently, we could not assess the relationship between surgeon volume and patient clinical outcomes.

## Conclusions

We performed a propensity-matched analysis to compare outcomes during hospitalization and 3 weeks after discharge as well as cost-effectiveness in a cohort of patients with lung neoplasms who underwent VATS as a two-day surgery or inpatient surgery. Although no significant differences were detected in the rates of medical events after discharge between the groups, the TSG was associated with a statistically significant reduction in mean total hospitalization expenses and some main components such as basic medical, nursing, drug, laboratory-related, and nonsurgical consumable costs. Our findings indicate that VATS can be safely conducted as a two-day procedure for lung neoplasms with cost-effectiveness in selected patients under the conditions of accurate preoperative evaluation, successful intraoperative assessment, and effective postoperative health care guidance.

## Supplementary Information

**Additional file 1.**

## Data Availability

The datasets used and/or analyzed during the current study are available from the corresponding author on reasonable request.

## Abbreviations

CT: Computed tomography
ERAS: Enhanced recovery after surgery
IQR: Interquartile range
ISG: Inpatient surgery group
LOS: Length of stay
SD: Standard deviation
TSG: Two-day surgery group
VAS: Visual analog pain scale
VATS: Video-assisted thoracoscopic surgery
