# Supplementary material for: Video-assisted thoracoscopic treatment as two-day surgery for lung neoplasms: a propensity-matched analysis
Source: BMC Cancer. 2022 Jul 30;22:832. doi: 10.1186/s12885-022-09938-x (PMC9338576; doi:10.1186/s12885-022-09938-x)
Supplement: Supplementary file 1 — Additional file 1. [file 12885_2022_9938_MOESM1_ESM.docx]

**Additional files**

Additional file 1. Total and Component Costs (¥) after Propensity Matching (1:1)

| Variable | ISG | TSG | Δ ISG-TSG (%) | *P* value |
| --- | --- | --- | --- | --- |
|  | 175 | 175 |  |  |
| Total costs | 36,815.6 | 33,410.9 | 3,404.7 (9.2) | <0.001 |
| General medical service fees | 317.9 | 114.1 | 203.8 (64.1) | <0.001 |
| General treatment fees | 884.4 | 460.4 | 424.0 (47.9) | <0.001 |
| Nursing fees | 618.4 | 279.9 | 338.5 (54.7) | <0.001 |
| Drug costs | 4,123.7 | 3,301.6 | 822.1 (19.9) | <0.001 |
| Material fees |  |  |  |  |
| For surgery | 14,559.7 | 14,293.6 | 266.1 (1.8) | 0.626 |
| For other treatment | 1,943.2 | 1,177.3 | 765.9 (39.4) | <0.001 |
| For examination | 82.5 | 65.5 | 17.0 (20.6) | <0.001 |
| Diagnostic-related fees |  |  |  |  |
| Laboratory | 2,302.3 | 1,872.8 | 429.5 (18.7) | <0.001 |
| Imaging | 1,829.2 | 1,807.1 | 22.1 (1.2) | 0.873 |
| Surgical treatment fees | 7,790.7 | 7,951.5 | -160.8 (-2.1) | 0.169 |

ISG: inpatient surgery group; TSG: two-day surgery group
